# Tissue Engineering in Neuroscience: Applications and Perspectives

**DOI:** 10.34133/bmef.0007

**Published:** 2023-01-16

**Authors:** Xiaoge Zhang, Fuyao Liu, Zhen Gu

**Affiliations:** ^1^ Liangzhu Laboratory, Zhejiang University Medical Center, Hangzhou 311121, China.; ^2^ Zhejiang Provincial Key Laboratory for Advanced Drug Delivery Systems, College of Pharmaceutical Sciences, Zhejiang University, Hangzhou 310058, China.; ^3^ Department of General Surgery, Sir Run Run Hospital, School of Medicine, Zhejiang University, Hangzhou 310016, China.; ^4^ MOE Key Laboratory of Macromolecular Synthesis and Functionalization Department of Polymer Science and Engineering, Zhejiang University, Hangzhou 310027, China.; ^5^ Jinhua Institute of Zhejiang University, Jinhua 321299, China.

## Abstract

Neurological disorders have always been a threat to human physical and mental health nowadays, which are closely related to the nonregeneration of neurons in the nervous system (NS). The damage to the NS is currently difficult to repair using conventional therapies, such as surgery and medication. Therefore, repairing the damaged NS has always been a vast challenge in the area of neurology. Tissue engineering (TE), which integrates the cell biology and materials science to reconstruct or repair organs and tissues, has widespread applications in bone, periodontal tissue defects, skin repairs, and corneal transplantation. Recently, tremendous advances have been made in TE regarding neuroscience. In this review, we summarize TE’s recent progress in neuroscience, including pathological mechanisms of various neurological disorders, the concepts and classification of TE, and the most recent development of TE in neuroscience. Lastly, we prospect the future directions and unresolved problems of TE in neuroscience.

## Introduction

The nervous system (NS) controls both physiological and pathological processes [1]. It consists of a complex neural network and coordinates the actions of numerous body parts *via* signal transmission, while simultaneously aiding the body in sensing and responding to external changes [2]. The central nervous system (CNS) and peripheral nervous system (PNS) make up the NS. The CNS, the core of the human NS, is composed of the brain and spinal cord [3,4]. Peripheral nerves (PN), located outside of the CNS, mainly connect various body parts to the CNS, which receives sensory signals from sensory nerve fibers and then responds and sends them to the target effectors *via* motor nerve fibers [5,6]. The CNS traumas, including traumatic brain injury (TBI) and spinal cord injury (SCI), affect millions of people worldwide annually and cost approximately US$406 billion [7]. The PNS injury is generally caused by various diseases, such as diabetes and autoimmune diseases [8,9]. Briefly, either CNS or PNS injury can disrupt the communication between brain tissue and target tissue, resulting in a loss of function, decreased productivity, and increased costs for social services. Therefore, regeneration of injured nerves is essential to facilitate their reconnection with distal target tissues for the purpose of restoring injured function.

**Fig. 1.**
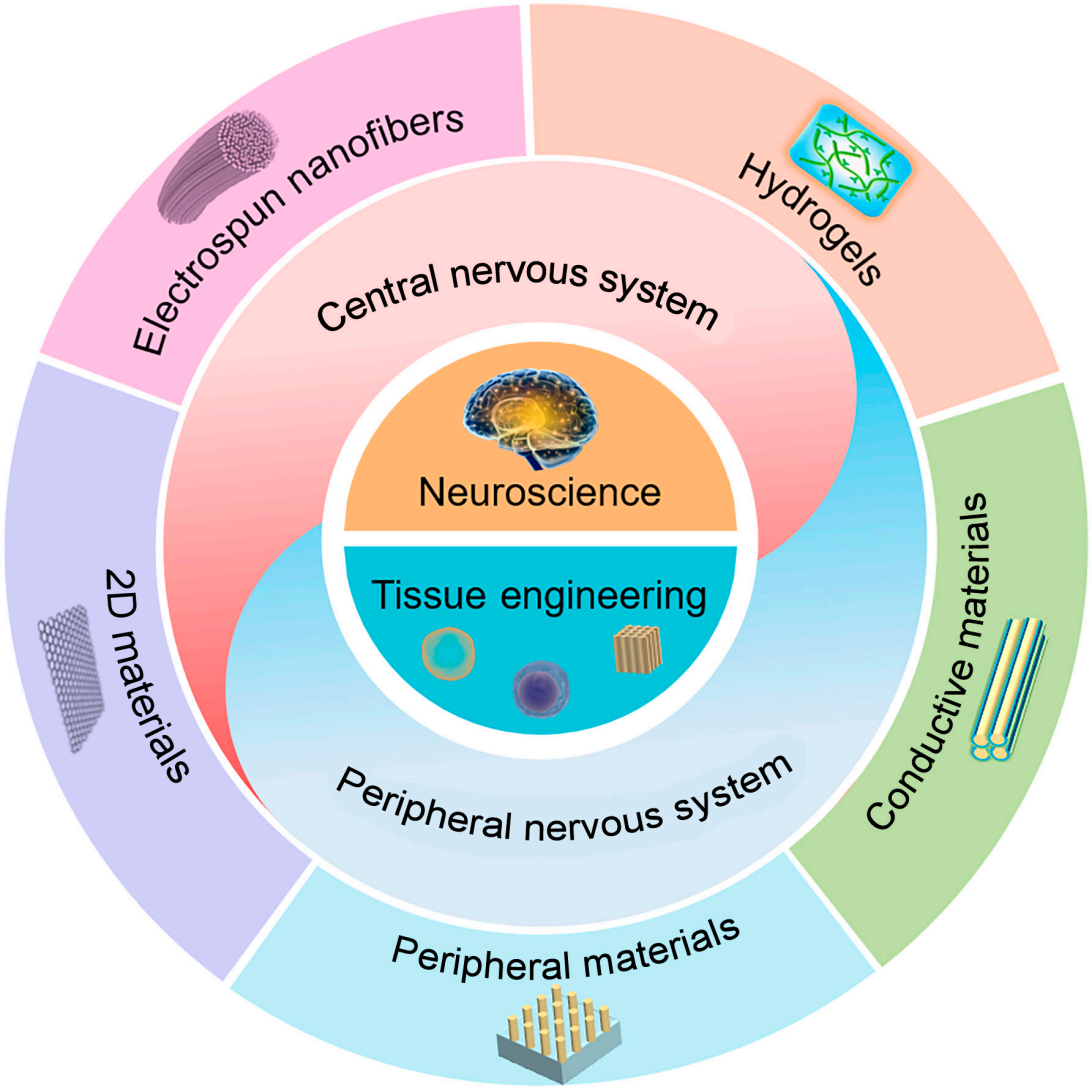
The applications of tissue engineering (TE) in neuroscience.

To address the issues of nerve regeneration, a growing number of techniques for repairing and treating nerve damage are being employed [10,11]. Surgery, drugs, cell therapy, exosomes, and tissue engineering (TE) scaffolds, are the most frequently employed treatment methods [12–15]. Among them, the rise and development of TE and regenerative medicine have brought a new approach for repairing and regenerating the damaged tissues and organs, thus transcending conventional treatment’s limitations. TE can produce grafts that mimic the natural extracellular matrix (ECM) structure and improve the nerve regeneration microenvironment through specific biochemical and topological signals, thereby promoting the CNS and PNS regeneration and damage repair. In this review, we selectively survey the pathogenesis of neurologic disorders, the most recent progress and advances in TE, and the main applications of TE regarding neuroscience (Fig. 1).

## Pathogenesis of Neurologic Disorders

The NS, composed of central and peripheral nerves, is the most important organ of the human body [1]. Neurologic disorders refer to the CNS or PNS with sensory, consciousness, movement, and other disorders as the main manifestations of the diseases, which mainly include Parkinson’s disease, Alzheimer’s disease, stroke, amyotrophic lateral sclerosis (ALS), Huntington’s disease, cerebellar atrophy, SCI, and TBI, among others.

The pathogenesis of CNS injury is a complex process primarily caused by primary and secondary injuries, which can result in temporary or permanent neurological deficits [16]. The primary injury is caused by external trauma to the brain or spinal cord. Secondary injuries can occur minutes to days after the initial injury, further damaging the CNS *via* molecular, chemical, and inflammatory cascades. In particular, the secondary inflammatory response is a key factor causing nerve damage. The activity of infiltrating macrophages and in situ activated microglia peaked on days 3 and 7 after injury, and the pro-inflammatory factors stimulated macrophages/microglia to polarize to the activated macrophages (M1 type) [17,18]. These macrophages/microglia release additional pro-inflammatory factors to exacerbate the damage. Similarly, pro-inflammatory factors can trigger the aggregation of astrocytes, resulting in the formation of neural scars that inhibit the regeneration of neural axons [19,20]. Besides, the inflammatory response can cause the destruction of the blood–brain barrier (BBB), worsen brain edema, and damage the CNS [21].

The PNS is composed of PN, which contains both myelinated and unmyelinated nerve fibers [4]. Thereinto, myelinated nerves consist of nerve fibers surrounded by Schwann cells (SCs), which provide insulating properties to facilitate the quick transmission of nerve signals. Most PNS damages are from direct mechanical damage, occasionally secondary to tumor resection. The PNS injuries could result in visible symptoms, such as numbness, sensory loss, neuropathic pain, and even motor dysfunction, affecting patients’ normal life in their professional and leisure activities. The PNS has an inherent capacity of repair and regeneration. The regenerate ability of PNS relies on the patient’s age, the manner of injury, especially the location of the injury, and the cell body of nerve cells.

The pathogenesis of these diseases, particularly CNS damage, is controversial and complicated. No conclusive mechanism exists at present. It is believed that inflammation and oxidative stress, metabolic disturbances, calcium channel dysfunction, mitochondrial disturbances, and neurotrophic factor (NF) deficiency are associated to these diseases [22,23].

## Tissue Engineering

TE emerged as a field of study in the 1980s and has continued to develop ever since [24]. Fung, a Chinese-American scientist, coined the term “tissue engineering” in 1984 [25]. Profs. Langer and Vacanti refined the definition of TE in 1993 as “the application of engineering and life sciences principles to the development of biological tissue or organ substitutes to restore, maintain, or enhance tissue and organ function” [26]. The basic principle of TE involves inoculating cells from human tissues onto bioscaffold, incubating them in the presence of external growth factors (GFs) and a suitable medium, and then transplanting the obtained specific tissues or organs into the injured area [27,28]. This technique could reduce the risk of immune rejection and secondary surgery, allowing for a more effective repair and treatment.

Currently, TE technology is advancing rapidly, and its research content and methods are continuously evolving [29–31]. Traditional TE concept has been continuously extended and broadened, and some neural tissues constructed using TE technology have achieved preliminary clinical applications [16]. Four crucial factors are often necessary for the regeneration of neural tissue: (a) bioscaffold, (b) seed cells, (c) GF, and (d) electrical stimulation (ES) [32].

### Bioscaffold

Bioscaffold is a 3-dimensional structure that can be transplanted inside the body and organically integrated with living cells. In the area of neural TE, the ideal bioscaffolds must possess the qualities listed below: (a) high biocompatibility, (b) good degradability, (c) excellent mechanical properties, and (d) good cell–interface relationship. Biomaterials are necessary for the fabrication of bioscaffolds in TE. Currently, biomaterials have evolved from inert or single materials to multifunctional bioactive materials for adapting to the body and prevent inflammation and immune rejection. With the continuous progress and development of biomaterials, the clinical application of bioscaffolds is increasingly extensive. According to the source, bioscaffold materials are mainly classified into natural and synthetic biomaterials [33–36].

Natural biological materials can be split into 2 generic groups: (a) biological tissues and their derivatives, such as blood vessels, skeletal muscles, human amniotic membrane, veins, and epineurium [37]; and (2) macromolecular substances extracted from living organisms, such as collagen, chitosan, hyaluronic acid (HA), alginate, gelatin, silk fibroin, and sericin. Compared to synthetic materials, natural biological materials possess superior biocompatibility, less toxicity, and enhanced cell adhesion and migration [38]. Collagen, of which 28 forms have been identified [39], is one of the most prevalent proteins of the ECM and presents in all connective tissues, including skin, bones, cartilage, tendons, and nerves [39]. It has been reported that collagen can support the adhesion and migration of nerve tissue cells, and its intermediate product (chitosan oligosaccharide) could also protect nerve cells and promote nerve regeneration [40,41]. Although collagen has various merits, such as low antigenicity, strong biocompatibility, high mechanical strength, and cross-linking capacity, it also has some drawbacks, including difficult sterilization and the inability to control space and degradation rate. HA, a glycosaminoglycan with nonsulfated, nonbranched, and linear specificity, is composed of repeating disaccharides (1,4-d-glucuronic acid, and 1,3-*N*-acetyl-glucosamine) and exists in extracellular tissues throughout the human body [42]. HA has been utilized successfully in TE, particularly neural tissue, for purposes such as proliferation, differentiation, and stimulating neurite outgrowth, thereby exhibiting great promise for PN regeneration [43–45]. Because of its high abundance in the neural system, particularly the CNS, HA is a considerably biocompatible material for creating scaffolds for neural tissue repair applications.

Synthetic biomaterials primarily refer to synthetic polymers and are split into 2 categories based on the degradability: (a) nondegradable polymers, such as silica gel and expanded polytetrafluoroethylene, and (b) degradable polymers, such as poly(lactic acid), poly(glycolic acid), and poly(lactic-co-glycolic acid). Since the nondegradable polymers exist in the body for a long time, causing foreign body reactions and compression of regenerated tissues, their clinical application is challenging. On the contrary, degradable polymers often have good biocompatibility and appropriate mechanical characteristics and have already been applied in PNS and CNS repair [46]. In addition, it is essential to strike a balance between the rate of material degradation during scaffold formation and the effectiveness of nerve regeneration [47,48].

### Seed cells

Seed cells, the “source of life”, are the foundation of TE research. The origin of seed cells involved in TE can be allogeneic autologous cells, allogeneic cells, and xenogeneic cells. There are 2 ways to obtain seed cells: (a) direct tissue biopsy technology from specific tissues or organs, and (b) in vitro directional induction and differentiation of stem cells [49].

#### Schwann cells

SCs are glial cells inside the PN system, being one of the most common seed cells employed in nerve TE [50,51]. SCs can maintain and hydrate nerves, as well as heal and regenerate the damaged PN. They can be created and repaired using scaffolds, or they can be used on their own to promote nerve regeneration. The transplantation of cultured SCs into injured PN has been demonstrated to facilitate their functional recovery [52]. After transplantation, SCs can form Bungner bands to assist the regenerated axons in traversing the defect site. In conjunction with the ECM, SCs also release several NFs that promote axon regeneration and myelination [51–54]. Therefore, SCs play an essential role in cell therapy for nerve regeneration. Even so, the extraction of SCs inevitably causes the peripheral nerve injury (PNI) at the donor site, resulting in complications [55,56], and it is still difficult to extract and culture sufficient SCs in a short period of time to meet therapeutic needs [50,57].

#### Stem cells

Because of their self-renewal and pluripotency abilities, stem cells are regarded as the best source of seed cells in engineering neural tissue [58]. Stem cells, in contrast to SCs, are plentiful in diversity and source. To effectively employ stem cells in neural TE, it is essential to select the right cell type, optimize the quantity and technique of transplantation, and apply exogenous factors to maximize cell survival, reduce tumorigenicity, increase therapeutic efficiency, and ensure safety [59,60].

Various cells, including embryonic mesenchymal stem cells (MSCs), umbilical cord MSCs, and olfactory ensheathing cells, have been induced to differentiate into Schwann-like cells to replace the in situ SCs lost due to injury [61]. However, ethical concerns, damage to the donor site, and a dearth of tissue sources limit the extraction of the aforementioned cells. Because of their easy availability, rapid proliferation, low immunogenicity, and multidifferentiation capacity, MSCs have become the focus of current cell-related therapy. Dezawa et al. [61] found that bone marrow mesenchymal stem cells (BMSCs) could be differentiated into Schwann-like cells in vitro by cultivation in the corresponding induction medium, and in vivo experiments supported the idea that transplanted Schwann-like cells could promote PN regeneration. However, the source of BMSCs is limited, and the tissue extraction process can be burdensome for patients [62]. Adipose-derived stromal cells are superior to BMSCs for replacing autologous SCs due to easy accessibility, reproducibility, relative safety, low damage, and a high cell acquisition rate. Despite the beneficial effects of stem cells on nerve regeneration, their use in neural TE remains controversial. Because of the differences between animal experimental models and the human internal environment, technical challenges, therapeutic response variations, and ethical considerations must be taken into account during the translation of stem cell therapy from the laboratory to the clinic [63–65]. Additionally, standardization of treatment guidelines, injection dosage, and administration methods are key factors in stem cell therapy that require additional research.

### Growth factors

In order to regulate cell proliferation, differentiation, migration, and gene expression, GF can bind to cell membrane receptors with high specificity and affinity [66–68]. GFs are derived from various sources, such as matrix-binding proteins attached to the ECM, soluble molecules secreted by cells and separated from the matrix by certain enzymes or proteases. GFs determine cell fate based on their chemical identity, concentration, duration, and context (presence and sequence of multiple factors) [69]. Certain GFs, for instance, promote angiogenesis, whereas others induce maturation and maintain the integrity of the established vascular system [70–72]. Meanwhile, GFs regulate communication between cells and their microenvironment and organs [73] and initiate subsequent transduction signals by recognizing specific receptors on the surface of target cells, whose expression levels partially determine the cell responsiveness. Notably, GFs are composed primarily of proteins, making it easy for high heat, high pressure, or organic solvents to compromise their functionality. However, these procedures are required for the production of scaffolding materials. Consequently, the preparation of GF-protecting materials must be considered thoughtfully.

### Electrical stimulation

**Fig. 2.**
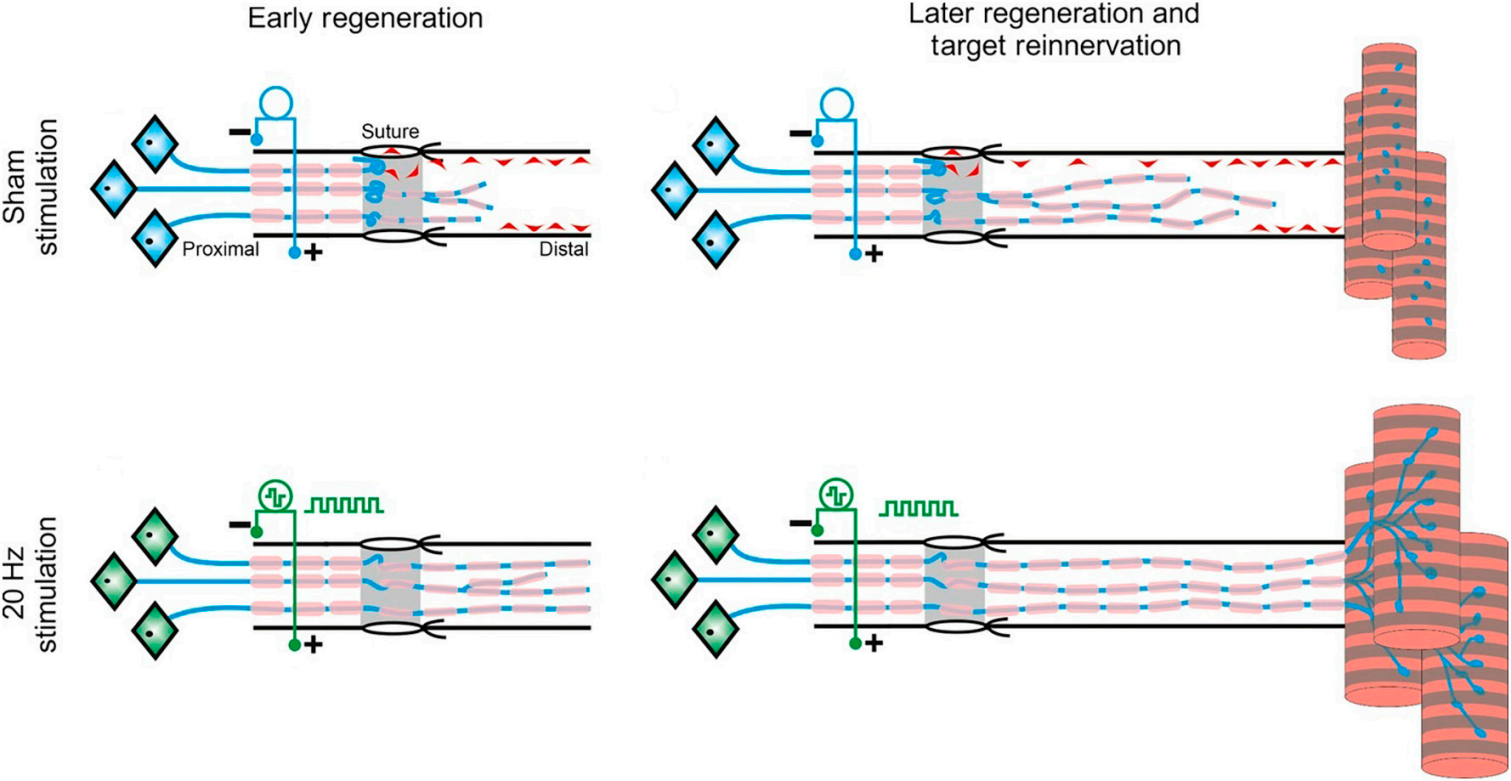
Electrical stimulation (ES) accelerates target reinnervation. ES (20 Hz, 1 h) accelerates the axons’ outgrowth at the surgical repair site of transected nerve stumps, resulting in accelerated target muscle reinnervation. Adapted from Ref. [76] with permission. Copyright 2016, Wiley-VCH.

Since Al-Majed et al. demonstrated that short-term low-frequency ES could promote axonal regeneration of transected nerves in 2000 [74], the field of electrical signal-promoting repair of nerve injury has garnered considerable scientific attention. ES provides several advantageous characteristics, including noninvasiveness, quick and reversible induction, and great spatiotemporal controllability [75]. ES can promote the generation of action potentials to enhance the cellular activity and the regeneration ability of damaged nerve axons, thereby facilitating the recovery of nerve function (Fig. 2) [76,77]. On the basis of the current direction and duration of action, ES is categorized as direct current, alternating current, pulsed current, and biphasic current stimulation. The magnitude and direction of direct current remain constant over time, whereas alternating current changes periodically in magnitude and direction. The pulsed current is short-lived, unidirectional, and bidirectional. ES can regulate the intracellular Ca^2+^ concentration in neurons and upregulate brain-derived NF and circulating AMP *via* a calcium-dependent mechanism, thereby promoting the upregulation of genes involved in tissue regeneration [78]. Besides, ES can induce the expression of nerve GF, thereby regulating the behavior of SCs and facilitating the remyelination of damaged nerve tissue [79]. Decades of preclinical research and recent prospective randomized clinical trials have demonstrated that ES is a promising nerve injury treatment.

## Application of TE in Neuroscience

The NS is crucial for communicating physiological processes of limbs and organs as well as controlling sensory and motor functions. Because of nerve injury, such as SCI, TBI, PNI, and neurodegenerative disorder, patients may experience a loss of sensory or motor function. With the development of TE in neuroscience area, implantable or injectable bioengineered biomaterials have been designed to restore the disturbed neural tissue architecture. Here, we emphasize bioengineered biomaterials, including electrospun nanofibers, supramolecular adhesive hydrogels, electroactive biomaterials, and 2-dimensional (2D) materials, that have been applied to neuroscience.

### Electrospun nanofibers

In the last 2 decades, electrospinning has been available in the production of nanofiber-based scaffolds for SCI treatment [80,81]. Although numerous fabrication techniques, such as self-assembly, super drawing, and phase separation, have been reported for nanofiber fabrication, electrospinning is still assumed to be one of the most versatile techniques for producing nanofibers with diameters ranging from a few to several hundred nanometers due to its simple processing, broad applicability, and enormous industrialization potential [82–85]. Because of their ability to imitate the fibrillar ECM’s intrinsic structure, nanofibers have been utilized extensively in the development of nerve guiding conduits [86]. These conduits aim to recapitulate the essential biological and structural features of the native ECM in order to provide a viable environment for guiding the regrowth and repair of transected nerves. Additionally, these fibers provide neural cells with favorable topographical and chemical cues and a route for the nutrients’ influx and waste efflux [87–89].

**Fig. 3.**
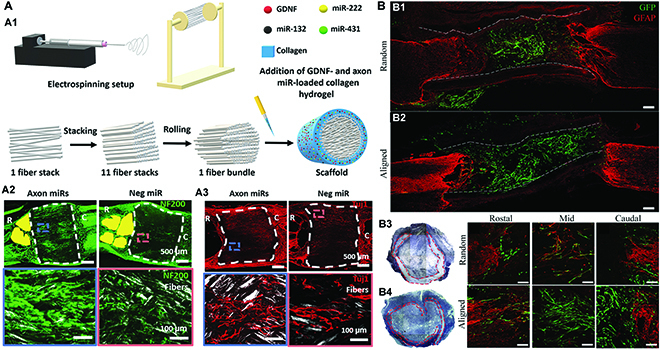
(A) Axon miRs promoted axon regeneration after SCI. (A1) Schematic diagram of the scaffold fabrication process. Representative fluorescence of NF200 (A2) and Tuj1 (A3) staining in rats at 12 weeks after SCI [92]. Produced with permission. Copyright 2021, Wiley-VCH. (B) Confocal fluorescence results of sagittal sections of spinal cords transplanted with GFP-SCs in random (B1) and aligned (B2) PVDF-TrFE conduits (outlined by white dashed lines, scale bars = 200 μm), and the tissue density in random (B3) and aligned (B4) conduits in 1-μm plastic sections (scale bars = 100 μm). Adapted from Ref. [93] with permission. Copyright 2017, Wiley-VCH.

Despite the fact that electrospun nanofibrous scaffolds alone can provide chemical cues and maintain structural stability due to their appropriate mechanical properties, their efficiency in promoting SCI regeneration and functional recovery is unsatisfactory. To further improve the SCI repair outcomes, other biochemical or biophysical factors, including bioactive component delivery, cell therapy, and external electrical or magnetic stimulation, have been introduced to generate a synergistic effect with the physical cues provided by electrospun scaffolds [80,81,90,91]. For instance, Zhang et al. [92] created a 3D scaffold with an aligned electrospun fiber bundle as the core part to physically direct the alignment of regenerating axons and collagen matrix as the sheath part to sustainably release glial cell-derived NF (GDNF) and several microRNAs (miRs), such as miR-132, miR-222, and miR-431 for biologically enhancing and regulating axon regeneration (Fig. 3A). Electrospun nanofibrous scaffolds could also create an instructional milieu for stimulating diverse cellular activities, making them excellent carriers for transporting seed cells in SCI therapy. As an example, SCs were transported *via* a conduit formed of electrospun polyvinylidene fluoride trifluoroethylene (PVDF-TrFE) nanofibers, which could greatly increase angiogenesis and axonal regeneration to promote SCI regeneration (Fig. 3B) [93]. Currently, electrospinning is utilized primarily for in vitro and in vivo experiments, with limited clinical applications. The next generation of electrospun neural scaffolds may combine bioactive molecules with nucleic acid therapy and establish a functional 3-dimensional structure to accelerate nerve growth recovery [94,95].

### Hydrogels

**Fig. 4.**
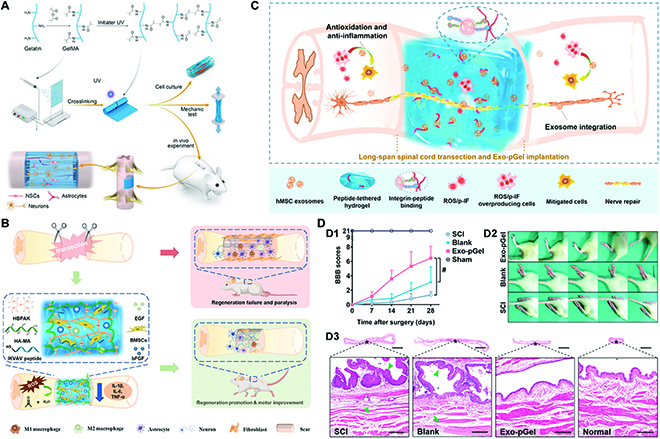
(A) Schematic diagram of the preparation process of GelMA hydrogel fibrous scaffold and the bionic scaffold for repairing the SCI. Adapted with permission from Ref. [99]. Copyright 2019, Wiley-VCH. (B) Schematic diagram of the BMSC-encapsulated reactive oxygen species (ROS)-scavenging hydrogel for SCI treatment. Reprinted from Ref. [103] with permission. Copyright 2023, KeAi Communications. (C) Illustration of Exo-pGel therapy for SCI. (D) Treatment of long-span SCI transection with Exo-pGel implantation and outcomes in functional recovery and urinary protection. BBB scores (D1) and typical records (D2) of animal walking gaits for 28-day-period treatments. (D3) HE staining of the bladder tissues (scale bars, 5 mm [top] and 200 μm [bottom]). Adapted from Ref. [105] with permission. Copyright 2020, American Chemical Society.

A network of cross-linked hydrophilic polymers is formed by water solubilization when physical or chemical cross-linking between hydrophilic macromolecules occurs [96]. Because of the great biocompatibility, high water content, porous structure, and modifiable mechanical strength, hydrogels are often applied to mimic the ECM in tissues. Moreover, hydrogels can serve as an excellent cellular scaffold and drug carrier for tissue and organ repair and regeneration [97,98]. Hydrogels can facilitate the delivery of mechanical stimuli to brain cells, which is essential for the regeneration of neural tissue (Fig. 4A and B) [99–103]. In a neuroregenerative environment, hydrogels primarily function as a local transport system for the delivery of medicines and other signaling molecules to the injury site, and then exert control over the host neural tissue by supporting cellular attachment, neurite development, proliferation, and differentiation [104]. Meanwhile, hydrogels can act as guiding cues to promote the development and repair of axonal and the restoration of SCI and TBI. Li et al. [105] encapsulated exosomes from human MSCs in a peptide-modified supramolecular sticky hydrogel (Exo-pGel) and employed it as a new implantation approach for treating SCI (Fig. 4C and D). However, there are a number of unanswered questions regarding hydrogel’s applications in TE. Hydrogel may form endogenous deposition in nerve, thereby impeding nerve regeneration. The excessive expansion of hydrogel materials may lead to secondary nerve damage by increasing local pressure [106].

### Electroactive biomaterials

**Fig. 5.**
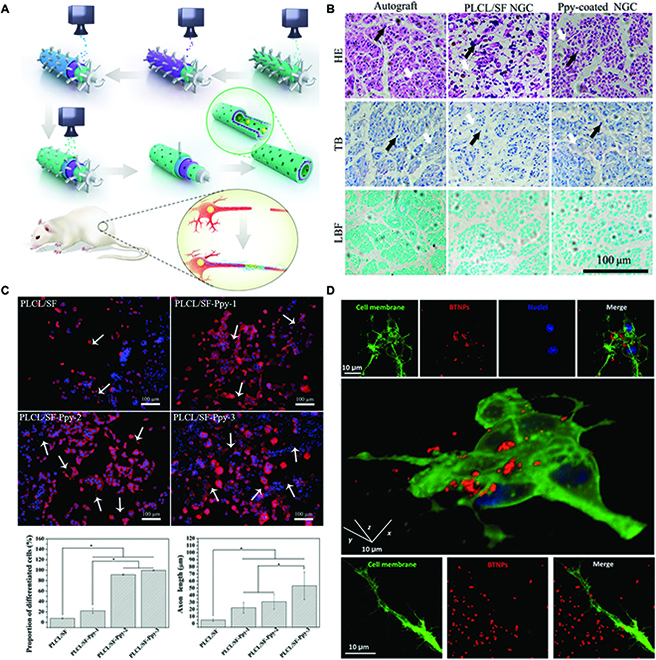
(A) Schematic illustration of graphene nerve conduit fabrication with layer-by-layer casting (LBLC) method. Adapted from Ref. [111] with permission. Copyright 2018, Nature Publishing Group. (B) Hematoxylin–eosin (HE), toluidine blue (TB), and luxol fast blue (LFB) staining of the generated nerves at the middle segment of the nerve guidance conduits (NGCs) (or autograft) after 12 weeks of implantation (the SCs were marked by black arrows and the ECM was marked with white arrows). Adapted from Ref. [116] with permission. Copyright 2019, Elsevier. (C) Immunofluorescence images of PC12 cells cultured on different materials. Adapted from Ref. [115] with permission. Copyright 2016, Royal Society of Chemistry. (D) Confocal fluorescence microscopy of BaTiO_3_ nanoparticles (BTNPs) associating to the neuronal plasma membranes. Adapted from Ref. [125] with permission. Copyright 2015, Nature Publishing Group.

Lately, electroactive biomaterials have been regarded as a new breed of intelligent biomaterials that can directly administer electrostimulation to target cells/tissues or change their features to adapt to the cell microenvironment. Various electroactive biomaterials, such as conductive and piezoelectric biomaterials, can replicate natural bioelectricity as a biophysical cue for modulating stem cell destiny and regenerative medicine [107–109]. For electrically excitable cells/tissues, electroactive materials facilitate charge transfer at the cell–substrate interface and govern cell–substrate or cell–cell interactions (Fig. 5A) [110–112]. Except for electrical capabilities, electroactive biomaterials can incorporate topological, chemical, and mechanical cues to suit the needs of particularly biological applications.

Cell activities including adhesion, proliferation, self-renewal or differentiation, and cellular signaling can be controlled on the conductive biomaterials [113,114]. Sun et al. demonstrated that ES (100 mV cm^–1^; 1 h per day) could further stimulate SC differentiation without neural GF, and neurite length increased with increasing polypyrrole concentration in the scaffold. On the basis of these findings, they developed a polypyrrole-coated nerve guidance conduit with nerve regeneration performance comparable to an autograft (Fig. 5B and C) [115,116].

Piezoelectricity is the generation of electricity from mechanical pressure [117,118]. Biomaterials with piezoelectric properties can create piezopotential to govern stem cell activity and destiny. Various piezoelectric biomaterials, including PVDF and its copolymer (PVDF-TrFE), have been used for neural differentiation and nerve regeneration [119–124]. Marino et al. [125,126] have proven that the produced piezopotential from BaTiO_3_ nanoparticles on cytomembranes and piezoelectric polymer scaffolds connected to cells could activate neuron-like cells or enhance the neuronal differentiation of stem cells in vitro under ultrasonic stimulation (Fig. 5D). In addition, certain tissue activities can be used to generate piezopotential, and the rational use of the interaction between piezoelectric biomaterials and cells, tissues, and organs is advantageous for their application in nerve TE [127,128].

### 2D materials

Since Andre Geim and Konstantin Novoselov discovered graphene more than a decade ago, 2D materials have been studied extensively in materials, biomedical research, chemistry, and nanoscience, exhibiting exceptional physical, electrical, and chemical capabilities [129]. Because of the surprising characteristic, plenty of 2D materials, including graphene, transition metal dichalcogenides, layered double hydroxides, transition metal carbides/nitrides (MXenes), metal-organic frameworks, covalent organic frameworks, and black phosphorus, have been well investigated in the last decades [130]. Except for the exceptional physical–chemical characteristics, 2D materials also demonstrate remarkable biosafety and degradability, enabling their use in TE [131,132]. Intriguingly, these nanomaterials facilitate the brain cells’ proliferation due to their high electrical conductivity, and the regulation of 2D materials on brain cell activities has been studied in recent years [133–135]. Therefore, 2D materials exhibited promising implementation prospects in scaffold construction in neurological disorders.

**Fig. 6.**
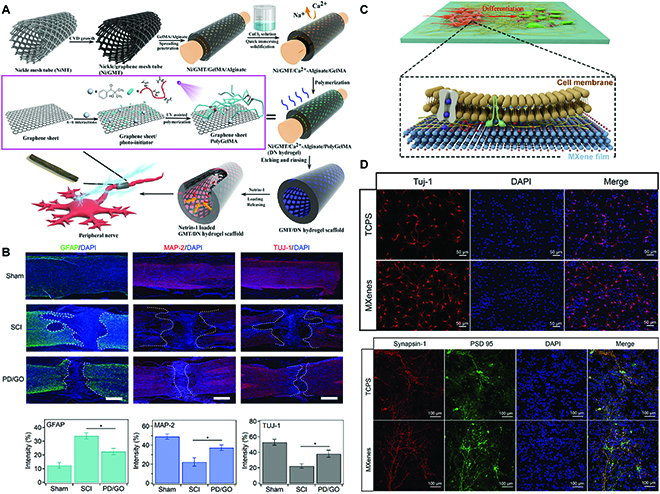
(A) Schematic illustration of netrin-1-loaded graphene mesh tube (GMT)/hydrogel nerve scaffold [136]. Produced with permission. Copyright 2021, American Chemical Society. (B) Immunofluorescence sections of the spinal cord repairing results [137]. Produced with permission. Copyright 2020, Elsevier. (C) Effects of the Ti_3_C_2_T*_x_*MXene film on the differentiation and maturation of neural stem cell (NSC)-derived neurons. (D) Representative fluorescent results of NSC differentiation and maturation on tissue culture polystyrene (TCPS) and Ti_3_C_2_T_x_MXene film. Adapted from Ref. [138] with permission. Copyright 2022, Elsevier.

For example, Huang et al. [136] designed a graphene mesh tube supported by a double-network hydrogel scaffold and further loaded with netrin-1 for PN regeneration (Fig. 6A). Besides, Zhang et al. [137] established a novel injectable 4-arm-polyethylene glycol-diacerein/graphene oxide hydrogel for SCI repair (Fig. 6B). Guo et al. [138] prepared a Ti_3_C_2_T_x_MXene film, which substantially promoted the neural differentiation ratio of neural stem cells compared to those on tissue culture polystyrene, resulting in neurons with longer neurites and an increased number of branch points and branch tips (Fig. 6C and D). Although numerous studies have demonstrated that 2D materials are biocompatible in vivo, their long-term safety has not been investigated. Future research should focus on the long-term cytotoxicity, biocompatibility, and metabolism of 2D materials in vivo, including the effects of the degradation rate of 2D materials on cell activity and the microscopic sizes of 2D materials on cell metabolism [139,140].

## Conclusions and Perspectives

Over the past year, impressive progress has been made in introducing the TE for neuroscience. However, the field of neurology still faces a multitude of challenges. Neurological disorders such as Alzheimer’s disease, Parkinson’s disease, epilepsy, brain stroke, ischemia, multiple sclerosis, and glioblastoma are regarded as the CNS’s most prevalent conditions, which are all associated with nonregeneration of neurons. Currently, there are no sufficient treatments that can fully restore the entire CNS. During the past several decades, a few promising animal experiments on CNS regeneration using novel biomolecules or mediators, such as *N*-methyl-D-aspartate (NMDA) receptor antagonists and monoclonal antibodies, have demonstrated improved neural network repair. However, when used in clinical studies for CNS regeneration, the same mediators demonstrated ineffective regenerative ability [141–143]. There are various causes for the delay in clinical transition. First of all, the CNS is not completely understood, and little is known about critical cellular and molecular processes and healing mechanisms, thereby impeding the development of novel medications. Secondly, the presence of the BBB, a high-selectivity semipermeable membrane that only enables low-molecular-weight and polar molecules (below 400 to 500 Da) to enter the CNS, drastically reduces the number of potential biomolecules for novel therapeutics. In recent years, brain–computer interfaces and optogenetics have been increasingly utilized as assistive technologies to aid in the regulation of neural signals and animal behavior. All these technologies could pass the BBB and directly affect the CNS.

Second, to preserve the scaffolds’ integrity until the regenerated tissue reaches maturity, the scaffold’s biodegradation rate must be controlled. Using 3D printing and digital light processing technology, it is quite probable to create a scaffold with a programmable interior structure nowadays. With the development of 3D printing technology, a vast array of biodegradable biomaterials will advance TE applications in nerve regeneration [144]. Finally, due to the vital impact of the body’s complex immune system, it is necessary to investigate and comprehend the interactions between biomaterials and host cells and tissues, as well as the complex bidirectional regulations.

In conclusion, TE offers great therapeutic promise for neurological disorders, but it has not yet met all of the patients’ needs, and the cellular and molecular processes involved in nerve regeneration require further study. As we improve our understanding of the mechanisms underlying NS diseases, a higher level of cross-disciplinary knowledge integration is required to realize TE clinical transformation and advancement in neuroscience.
